# Leukocyte-derived microparticles and scanning electron microscopic structures in two fractions of fresh cerebrospinal fluid in amyotrophic lateral sclerosis: a case report

**DOI:** 10.1186/1752-1947-6-274

**Published:** 2012-09-03

**Authors:** Anne C Zachau, Mikael Landén, Fariborz Mobarrez, Rolf Nybom, Håkan Wallén, Lennart Wetterberg

**Affiliations:** 1 Department of Neurology, Karolinska University Hospital, Stockholm, Sweden; 2 Karolinska Institutet, Department of Medical Epidemiology and Biostatistics, Stockholm, Sweden; 3 Institute of Neuroscience and Physiology, The Sahlgrenska Academy at Gothenburg University, Gothenburg, Sweden; 4 Karolinska Institutet, Department of Clinical Sciences, Danderyd Hospital, Division of Cardiovascular Medicine, Stockholm, Sweden; 5 Karolinska Institutet, Department of Neuroscience, Stockholm, Sweden; 6 Karolinska Institutet, Department of Clinical Neuroscience at St. Göran, Stockholm, Sweden

**Keywords:** Amyotrophic lateral sclerosis, Antibodies, Cerebrospinal fluid, Complementary methods, Electromyography, Flow cytometry, Microparticles, Phosphatidylserine, Scanning electron microscopy

## Abstract

**Introduction:**

Amyotrophic lateral sclerosis is a progressive neurodegenerative disorder characterized by degeneration of motoneuron cells in anterior spinal horns. There is a need for early and accurate diagnosis with this condition. In this case report we used two complementary methods: scanning electron microscopy and fluorescence-activated cell sorting. This is the first report to our knowledge of microparticles in the cerebrospinal fluid of a patient with amyotrophic lateral sclerosis.

**Case presentation:**

An 80-year-old Swedish man of Caucasian ethnicity presented to our facility with symptoms of amyotrophic lateral sclerosis starting a year before his first hospital examination, such as muscle weakness and twitching in his right hand progressing to arms, body and leg muscles. Electromyography showed classical neurophysiological findings of amyotrophic lateral sclerosis. Routine blood sample results were normal. A lumbar puncture was performed as a routine investigation and his cerebrospinal fluid was normal with regard to cell count and protein levels, and there were no signs of inflammation. However, scanning electron microscopy and fluorescence-activated cell sorting showed pronounced abnormalities compared to healthy controls. Flow cytometry analysis of two fractions of cerebrospinal fluid from our patient with amyotrophic lateral sclerosis was used to measure the specific binding of antibodies to CD42a, CD144 and CD45, and of phosphatidylserine to lactadherin. Our patient displayed over 100 times more phosphatidylserine-positive microparticles and over 400 times more cell-derived microparticles of leukocyte origin in his cerebrospinal fluid compared to healthy control subjects. The first cerebrospinal fluid fraction contained about 50% more microparticles than the second fraction. The scanning electron microscopy filters used with cerebrospinal fluid from our patient were filled with compact aggregates of spherical particles of lipid appearance, sticking together in a viscous batter. The quantitative increase in scanning electron microscopy findings corresponded to the flow cytometry result of an increase in leukocyte-derived microparticles.

**Conclusions:**

Microparticles represent subcellular arrangements that can influence the pathogenesis of amyotrophic lateral sclerosis and may serve as biomarkers for underlying cellular disturbances. The increased number of leukocyte-derived microparticles with normal cell counts in cerebrospinal fluid may contribute to the amyotrophic lateral sclerosis inflammatory process by formation of immune complexes of prion-like propagation, possibly due to misfolded proteins. The two complementary methods used in this report may be additional tools for revealing the etiology of amyotrophic lateral sclerosis, for early diagnostic purposes and for evaluation of clinical trials, long-term follow-up studies and elucidating the pathophysiology in amyotrophic lateral sclerosis.

## Introduction

Amyotrophic lateral sclerosis (ALS) has previously been described as a neurodegenerative disorder of upper and lower motor neurons, involving the cerebral cortex, brainstem and spinal cord, with progressive muscle weakness. However, recently, new evidence shows a more widespread degeneration, including cognitive and/or behavioral impairment. There is a clear association between ALS and frontotemporal lobe dementia (FTD). In both diseases a common protein can be found in TAR deoxyribonucleic acid (DNA)-binding protein 43 (TDP-43) in intra-neuronal inclusions [1]. Despite more than a hundred years of research no etiology has been found to this devastating disease; many theories have been put forward, but the pathogenesis is still unknown.

In 2012 the European Federation of Neurological Societies (EFNS) task force summarized the evidence for the diagnosis and management of ALS and made the following recommendations: ‘Patients with symptoms suggestive of ALS should be assessed as soon as possible by an experienced neurologist. Early diagnosis should be pursued’.

An accurate diagnosis is critical. ALS largely remains a clinical diagnosis based on signs of upper and lower motor neuron degeneration. Supporting electrophysiological studies such as electromyography (EMG) and exclusion of other etiologies using neuroimaging such as magnetic resonance imaging (MRI), are used routinely, as are clinical laboratory tests [2]. However, there is initially a risk of a misdiagnosis [3]. This makes the search for and finding of a biomarker for ALS all the more urgent. Some have been proposed, but there is still a lack of ALS-specific ones [4].

Patients with schizophrenia [5] and bipolar disorders [6] have previously been shown to display spherical micro-sized particles in their cerebrospinal fluid (CSF) although the disease symptoms in psychosis are supposed to mainly affect brain function. In the present study we opted to use two complementary techniques, fluorescence-activated cell sorting (FACS) [7] and scanning electron microscopy (SEM) [5] in a patient with neurological issues with a robust somatic disease known to engage the central nervous system (CNS) motor system, where death of neurons occurs in the anterior spinal cord more adjacent to the lumbar cerebrospinal fluid than in diseases with cognitive symptoms.

## Case presentation

An 80-year-old Swedish-born Caucasian man was referred to us from a private neurologist with a diagnosis of possible ALS six months ago. His mother died from ALS in her 80s, but no other relatives are known to have had this disease. Until recently our patient had been physically active, including participation in marathon running and cross-country skiing. Two years ago he had experienced a cardiac arrest but was successfully resuscitated. Aside from that, he had been in good health. About one year ago he started to experience a progressive weakness of his right hand and arm. During the same period, cognitive deterioration and personality changes appeared.

A neurophysiologic investigation including nerve conduction studies (NCS) and EMG were performed prior to his first hospital visit, including all four extremities, proximally and distally. The NCS showed normal sensory and motor conduction velocities. The EMG showed spontaneous denervation activity, fasciculations and neurogenic motor unit aberrations including polyphasia and reduced interference within all examined muscles (arms and legs, both proximal and distal). The conclusion of the neurophysiologist was that of a general motor neuron disease. The neurological examination showed a profound weakness of the right hand, a slight weakness of the left hand, and muscle atrophies in the hands, arms and shoulders. Fasciculations were widely spread over arms, shoulders, thorax and legs. The deep tendon reflexes were normal and both plantar reflexes reacted normal. The pain and touch sensations were normal. An MRI scan of the spinal canal showed no pathologic lesions of the spinal cord.

A routine analysis of the CSF was normal with regard to cell count and protein concentration, and there were no signs of ongoing inflammation. The serum and CSF antibody titers for *Borrelia* bacteria were normal. Routine blood sample results were normal (complete blood count, clinical biochemistry tests including liver, kidney and muscle, thyroid function tests, and electrolytes).

After receiving the results of these investigations, our patient and his wife were informed about the diagnosis of ALS, which was based on the following evidence: (1) clinical signs of lower motor neuron involvement in three regions (arms, thorax, and legs); (2) signs of upper motor neuron involvement (preservation of deep tendon reflexes (arms and legs); (3) electromyographic signs of denervation, fasciculations and polyphasia within all examined muscles, (4) absence of neuroimaging pathology; and (5) absence of evidence of other diseases.

According to the El Escorial Criteria [2] a ‘clinically probable ALS’ was suggested (evidence of upper motor neuron involvement was demonstrated in two regions).

An experienced neurologist performed the lumbar puncture with our patient in a sitting position. The skin in the lumbar region was washed with sterile cotton swabs before the puncture. A fine disposable needle of Quincke type, Becton, Dickinson and Company; 0.7 × 75mm (BD AB, Stockholm, Sweden) was used. The needle was inserted in the vertebral L3 to L4 inter-space and one trial was sufficient for CSF to flow. The very first two drops (100μL) of CSF were allowed to drip directly onto a polycarbonate filter, which was immediately vacuum dried by a special bedside pump and gold plated for microscopic examination. The following 200μL were collected straight in a sterile test tube for immediate FACS analysis of cell-derived microparticles (MP) in the fresh CSF. In summary, we tested the first 100μL of CSF with SEM followed by 200μL CSF for FACS, then 100μL of a second fraction of CSF for SEM, again followed by 200μL of CSF for FACS.

The collection of CSF from healthy subjects was part of an ongoing study approved by the Regional Ethics Committee in Stockholm (no. 2009/1221-32) and conducted in accordance with the latest Helsinki Protocol. Our patient and all control subjects consented orally and in writing to participate in the study.

### *Electron Microscopy*

**Figure 1:**
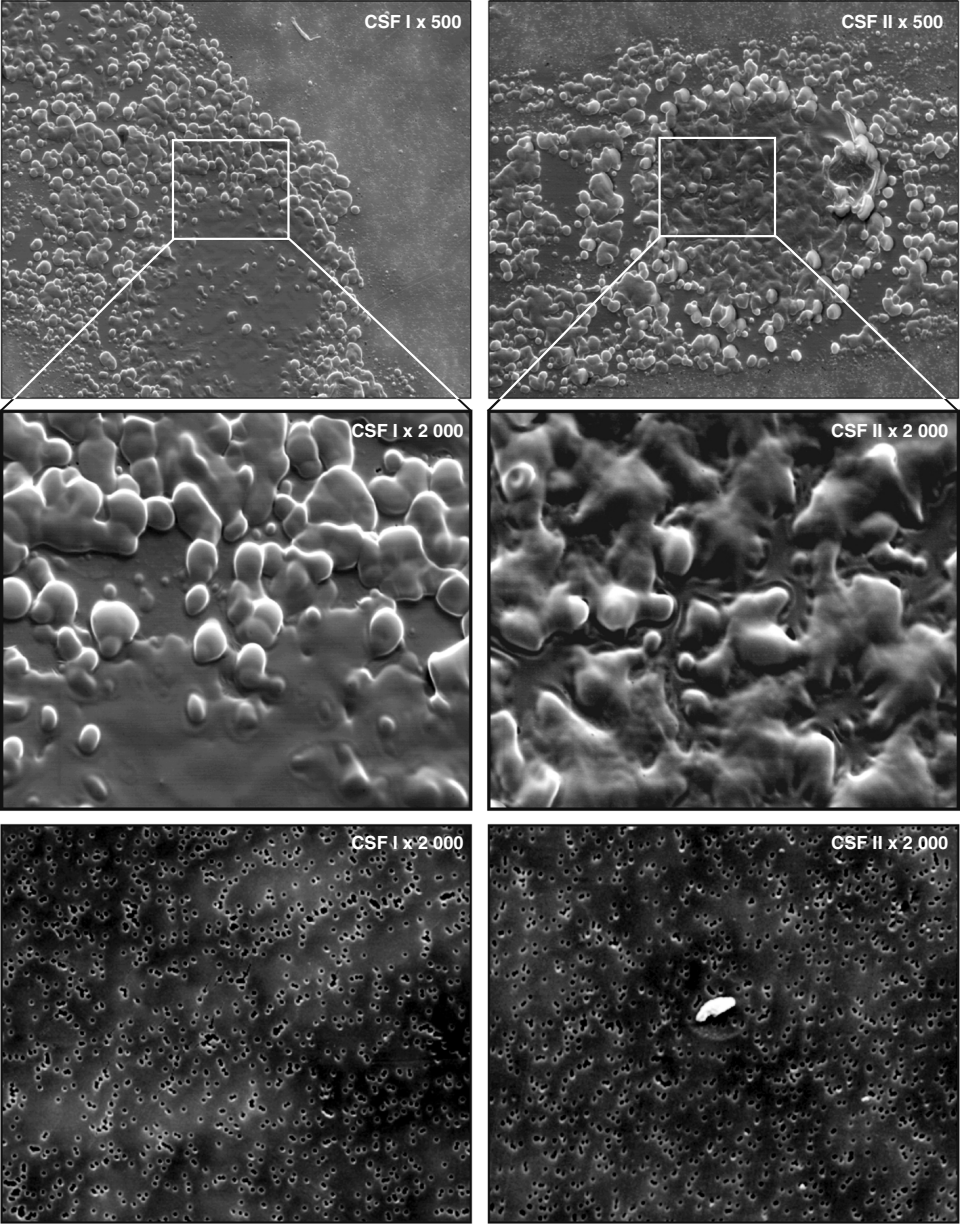
**Scanning electron microscopy (SEM) of two fractions of cerebrospinal fluid (CSF) from our patient with amyotrophic lateral sclerosis (ALS) and a control subject.** Cerebrospinal fluid was dripped onto the surface of a polycarbonate filter with 0.6μm pores (visible in the images and can be used for size reference). A total of 100μL of cerebrospinal fluid was used from our patient, and 200μL from the healthy control subject. Although only half the volume of control cerebrospinal fluid was used for our patient, the amyotrophic lateral sclerosis filters are filled with compact aggregates of spherical particles of lipid appearance, sticking together in a viscous batter. This process can be clearly seen in the image from our patient’s sample in the upper left corner (CSF I × 500) where the subcellular structures are closely attached, leaving the remaining filter free of aggregates. Control filters (CSF I × 2000) and (CSF II × 2000) are free of particles except a tiny skin flake in the middle of the control filter (CSF II × 2000).

For controls 200μL of the first 0.6mL fresh CSF aliquot and 200μL of the following 12mL of fresh CSF were allowed to drip directly on the surface of a polycarbonate filter (Nuclepore, Inc., Pleasanton, CA, USA) with 0.6μm pores. The polycarbonate filters were specially prepared by GP Plastic AB (Gislaved, Sweden) and supplied by Sempore AB (Stockholm, Sweden). The filter was fitted to an airtight device designed with flow channels, which allowed CSF particles to stream to the center of the filter when vacuum suction was applied from below. The pores of the filters were 0.6μm, ensuring that all particles with larger diameter stayed on the filter (see Figure 1). All remaining structures in the CSF were thus concentrated in the center of the filter during drying by vacuum suction. When the filters were completely dried after about two minutes of vacuum suction, they were subsequently coated in a JEOL JFC-1200 Fine Coater (JEOL Tokyo, Japan) for two minutes with ionized gold to a thickness of 40Å. The first and the second fractions of CSF were handled in a similar way in preparation for SEM, and all 17 healthy control subject samples were treated in a similar way to the sample from our patient.

The SEM method used in the study has earlier detected human immunodeficiency virus in CSF [8]. The total area of each filter with a diameter of 1cm was examined using a SEM microscope (Philips High Resolution SEM 515, Philips Electronic Instruments, Eindhoven, The Netherlands). The peripheral area outside the center was mostly free of structures. Two SEM images of the central areas of the filters were enlarged (×500 and × 2000).

### *Measurement of microparticles*

The CSF samples were analyzed fresh within an hour of collection. A total of 20μL of the sample was incubated for 20 minutes in the dark with phalloidin-Alexa-660 (Invitrogen, Paisley, UK), lactadherin-FITC (Haematologic Technologies Inc., Vermont, USA), CD42a-PE (BD, New Jersey, USA), CD144-APC (AH diagnostics Ltd., Stockholm, Sweden) and CD45-PC7 (Beckman Coulter, Inc. Brea, California, USA). MPs were measured on a Beckman Coulter Gallios™ flow cytometer (Beckman Coulter, Inc. Brea, California, USA). The MP-gate was determined using megamix beads (BioCytex, Marseille, France), which is a mix of three beads with diameters of 0.5μm, 0.9μm and 3μm, respectively. MPs were defined as particles less than 1.0μm in size, negative to phalloidin (in order to exclude cell membrane fragments [7]) and positive to lactadherin (which binds to phosphatidylserine). Later the MPs were sorted into particles positive for CD42a (platelet origin), CD144 (endothelial origin) or CD45 (leukocyte origin). Conjugate isotype-matched immunoglobulin (IgG1-FITC, IgG1-PE, IgG1-APC and IgG1-PC7) with no reactivity against human antigens was used as a negative control to define the background noise in the cytometry analysis. The results are presented as number of events in 45 seconds in the MP-gate during 45 seconds of measurement.

### *Findings of FACS and SEM*

**Figure 2:**
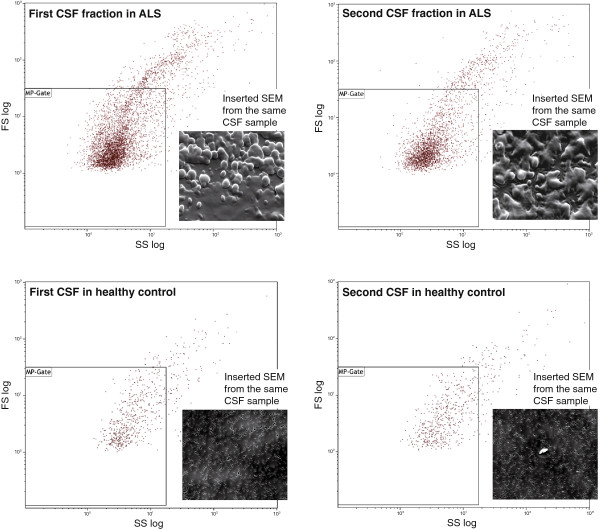
**Flow cytometry of two fractions of cerebrospinal fluid (CSF) from our patient with amyotrophic lateral sclerosis (ALS) and a healthy control subject.** Microparticles (MP), considered to have a size less than 1.0μm, were discriminated by their forward (FS) and side scatter (SS) characteristics. These microparticles were then analyzed for specific binding of lactadherin (detects phosphatidylserine (PS)) and antibodies towards CD42a (platelet microparticles), CD144 (endothelial microparticles) and CD45 (leukocyte microparticles) as presented in Table 1. The scanning electron microscope (SEM) photographs (enlarged × 2000) of the same fractions of cerebrospinal fluid are inserted to the right of the microparticle gates and further described in Figure 1.

**Figure 3:**
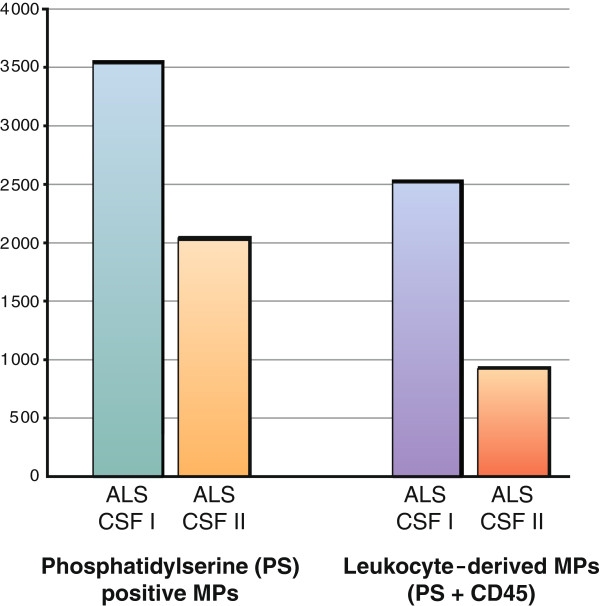
**Flow cytometry of phosphatidylserine (PS) and CD45 antibodies in two fractions of cerebrospinal fluid (CSF) in our patient with amyotrophic lateral sclerosis (ALS).** Number of events (that is, number of microparticles [MPs]) detected during 45 seconds of measurement are shown on the y-axis. See Figure 2 for further information on methodology.

The results of the two complementary methods, FACS and SEM, were available within two hours of the lumbar punctures (Figure 2). Our patient’s samples displayed more than 100 times the number of microparticles in CSF, measured as events of phosphatidylserine (PS)-positive MPs compared to the CSF samples from four healthy controls (Table 1, Figure 3). Most cell-derived MPs in CSF were positive for CD45 and thus of leukocyte origin (leukoctye-derived microparticles; LMPs). Our patient had almost 400 times more LMPs in his CSF than the four healthy control subjects had in theirs. The platelet (CD42a)-derived and endothelial cell-derived (CD144) MPs were low and within similar ranges as the healthy control subjects. The number of PS-MP and LMP in the first fraction of CSF was about 50% higher than the MP events in the second CSF fraction (Figure 3, Table 1). The scanning electron microscopic evaluations of CSF of our patient showed many spherical particles compared to no particles seen in the CSF from the 14 healthy control subjects, 10 of whom were matched for age to our patient and used only for the SEM analysis (Figure 1).

**Table 1:** Microparticles (MPs) in cerebrospinal fluid (CSF) from our patient with amyotrophic lateral sclerosis (ALS) and from four healthy controls, respectively

**Individuals**	**CSF fraction**	**Phosphatidylserine**	**CD42a**	**CD144**	**CD45**
Patient	CSF I	3544	4	4	2526
Patient	CSF II	2009	5	13	1873
Controls	CSF I	32	9	2	6
Controls	CSF II	31	8	1	7

Flow cytometry was used to detect microparticles from platelets (CD42a), endothelial cells (CD144) and leukocytes (CD45) in the first (CSF I) and second fraction (CSF II) of cerebrospinal fluid. The results are presented as number of microparticles detected in the cerebrospinal fluid. See Figure 2 for further information on the method.

## Discussion

Microparticles carry identity proteins and bioactive molecules from the parental cell. Their detection and identification in the CSF may be considered as a direct indicator of activation or damage from specific cells or tissues. CSF was selected in the present case for its closeness to CNS pathology in the motor neurons in the anterior spinal horns in amyotrophic lateral sclerosis. The clearance of apoptotic cells by phagocytes is an efficient process. However, even in tissues that are known to contain a large fraction of cells undergoing apoptosis (such as the bone marrow), it is difficult to detect apoptosis by traditional methods because the apoptotic cells are rapidly engulfed by macrophages. The exposure of phosphatidylserine that occurs during apoptosis is the best-studied macrophage recognition signal, and several receptors have been identified in macrophages that mediate apoptotic cell clearance by binding to phosphatidylserine on apoptotic cells directly or indirectly.

The number of SEM structures, which are likely emerging from the fragments of the destruction of motor neurons from the frontal spinal horn, corresponds to the events of MPs calculated as PS-MPs in the MP gate (see Figure 2). The destroyed lipid cell membranes of the leukocytes are expected to form different sizes of spherical particles when they aggregate and accumulate in the CSF. The amount of particles in the CSF of our patient seen under the microscope in the first CSF fraction compared to the second CSF fraction indicates that there was a gradient of the number of particles in the CSF (Figure 3). This finding corresponds to the number of MPs measured with the FACS technique. The gradient in the number of particles may be explained by the anatomy of the peripheral cerebrospinal fluid outflow pathway [9].

In general terms microglial phagocytosis of dead or dying neurons can be beneficial by preventing the release of damaging inflammatory intracellular components. However, there is evidence that under certain conditions microglia can also phagocytose viable neurons, thus initiating their death. Such phagocytic cell death may result from exposure of PS as an ‘eat-me’ signal (for example, via receptor brain-specific angiogenesis inhibitor 1; BAI 1) destroying otherwise viable neurons. The neuronal cell death in ALS may start as a result of a variety of alleged etiological factors such as genetic vulnerability or different toxic substrates, which may spread neuronal phagocytosis by activated microglia [10].

In our patient lumbar puncture was performed before the diagnosis of ALS was finally verified. We found abundant LMPs in his CSF. The LMPs may have originated from neutrophils, monocytes or macrophages, and lymphocytes. MPs express markers from their parental cells and harbor membrane and cytoplasmic proteins as well as bioactive lipids implicated in a variety of mechanisms, maintaining or disrupting homeostasis. Both pro-inflammatory and anti-inflammatory processes can be affected in several ways by LMPs to ensure an appropriate inflammatory response. LMPs have also been shown to play a dual role in the endothelium by either improving the endothelial function or inducing an endothelial dysfunction [11]. We did not find any MPs in CSF derived from endothelial cells in our patient. Numbering microparticles, including antibodies for LMPs, might be useful in detecting early signs of ALS. Additional work is of course needed to further investigate the overall role of LMPs in ALS.

There is still no consensus on the etiology of ALS. Researches show evidence of intoxication by heavy metals, environmental and occupational causes, genetic mutations, for example, of superoxide dismutase 1 [12] or of d-amino acid oxidase with accumulation of d-serine in the spinal cord [13], and viral infections. There are also signs of more general apoptosis in patients with ALS reflected as increase of caspase 9 in serum [14]. Due to this great diversity of possible causative agents for ALS, it is necessary to elucidate many possible etiologies for a better approach to patients, promoting preventive programs for the disease, optimizing functions and improving the quality of life of the patients.

The two complementary methods presented in this case report might help to diagnose the first signs of pathophysiology in the CSF in larger sets of individuals with high risk of developing ALS. Including more antibodies as FACS markers in CSF studies may also be useful in differentiating clinical subtypes of patients with ALS as well as in diagnosing other neuropsychiatric diseases. One advantage with the two matching methods is that the results of both procedures may be available within a short time (one hour) after the LP procedure. The rapid analysis, which may easily be repeated in clinical trial, may also be of particular use for researchers who develop and test new treatment forms for patients with ALS.

## Conclusions

Our 80-year-old patient with ALS symptomatology showed 100 times more phosphatidylserine-positive MPs and 400 times more cell-derived microparticles of leukocyte origin in fresh CSF compared to healthy control subjects. The increased number of MPs may contribute to the ALS inflammatory process by formation of immune complexes and interact with prion-like propagation of misfolded proteins in neural cells [15]. MPs thus represent subcellular arrangements in ALS that might serve as biomarkers for the disease. The two complementary methods SEM and FACS used in this report may be additional tools for rapid early diagnostic purposes, in evaluating clinical trials and in follow-up studies of patients with ALS, and for elucidating the pathogenesis of ALS.

## Consent

Written informed consent was obtained from the patient for publication of this case report and any accompanying images. A copy of the written consent is available for review by the Editor-in-Chief of this journal. Additionally, the collection of cerebrospinal fluid from healthy subjects was part of an ongoing study approved by the Regional Ethics Committee in Stockholm (no. 2009/1221-32) and conducted in accordance with the latest Helsinki Protocol. Our patient and all control subjects consented orally and in writing to participate in the study.

## Competing interests

None of the funding sources were involved in the preparation of or decision to submit this manuscript. RN is the inventor and patent owner of the special airtight device of the filter from Sempore, Stockholm, Sweden, used in this study.

## Authors’ contributions

ACZ performed the clinical examination, the lumbar puncture and interpreted the data from our patient. ML and LW were responsible for CSF sampling of the healthy control subjects. FM and HW performed the flow cytometric analysis and RN the scanning electron microcopy. FM and LW were major contributors in writing the manuscript. All authors read and approved the final manuscript.
